# N-terminal truncations on L1 proteins of human papillomaviruses promote their soluble expression in *Escherichia coli* and self-assembly in vitro

**DOI:** 10.1038/s41426-018-0158-2

**Published:** 2018-09-26

**Authors:** Minxi Wei, Daning Wang, Zhihai Li, Shuo Song, Xianglin Kong, Xiaobing Mo, Yurou Yang, Maozhou He, Zhongyi Li, Bo Huang, Zhijie Lin, Huirong Pan, Qingbing Zheng, Hai Yu, Ying Gu, Jun Zhang, Shaowei Li, Ningshao Xia

**Affiliations:** 1 State Key Laboratory of Molecular Vaccinology and Molecular Diagnostics, School of Life Sciences, Xiamen University, 361102 Xiamen, China; 2 National Institute of Diagnostics and Vaccine Development in Infectious Disease, School of Public Health, Xiamen University, 361102 Xiamen, China; 3 Xiamen Innovax Biotech Company, Ltd, 361022 Xiamen, China

## Abstract

Human papillomavirus (HPV) is the causative agent in genital warts and nearly all cervical, anogenital, and oropharyngeal cancers. Nine HPV types (6, 11, 16, 18, 31, 33, 45, 52, and 58) are associated with about 90% of cervical cancers and 90% of genital warts. HPV neutralization by vaccine-elicited neutralizing antibodies can block viral infection and prevent HPV-associated diseases. However, there is only one commercially available HPV vaccine, Gardasil 9, produced from *Saccharomyces cerevisiae* that covers all nine types, raising the need for microbial production of broad-spectrum HPV vaccines. Here, we investigated whether N-terminal truncations of the major HPV capsid proteins L1, improve their soluble expression in *Escherichia coli*. We found that N-terminal truncations promoted the soluble expression of HPV 33 (truncated by 10 amino acids [aa]), 52 (15 aa), and 58 (10 aa). The resultant HPV L1 proteins were purified in pentamer form and extensively characterized with biochemical, biophysical, and immunochemical methods. The pentamers self-assembled into virus-like particles (VLPs) in vitro, and 3D cryo-EM reconstructions revealed that all formed *T* = 7 icosahedral particles having 50–60-nm diameters. Moreover, we formulated a nine-valent HPV vaccine candidate with aluminum adjuvant and L1 VLPs from four genotypes used in this study and five from previous work. Immunogenicity assays in mice and non-human primates indicated that this HPV nine-valent vaccine candidate elicits neutralizing antibody titers comparable to those induced by Gardasil 9. Our study provides a method for producing a nine-valent HPV vaccine in *E. coli* and may inform strategies for the soluble expression of other vaccine candidates.

## Highlights

• N-terminal truncations promote the soluble expression of HPV L1 proteins in *E. coli* and their self-assembly of *T* = 7 icosahedral particle in vitro

• An HPV 9-valent vaccine candidate was formulated with *E. coli*-derived HPV 6, 11, 16, 18, 31, 33, 45, 52, and 58 VLPs, and conferred comparable immunogenicity with Gardasil 9

## Introduction

Human Papillomavirus (HPV) is a small, non-enveloped epitheliotropic DNA virus associated with multiple human diseases, such as benign verrucae vulgares (common wart), condylomata acuminata (genital warts), and malignancies of the cervix, vulva, anus, and penis^1,2^. Nearly 200 HPV types have been identified to date, with over 15 high-risk types closely linked to cervical cancer and other epithelial tumors^3,4^. Cervical cancer is one of the most common types of female cancers, of which 88% are caused by HPV types 16, 18, 31, 33, 45, 52, and 58^4–6^. Specifically, HPV 33, 45, 52, and 58 account for 15% of cervical cancers worldwide^7,8^. HPV 6 and HPV 11, which are considered as low-risk HPV subtypes, are usually not connected with cancer but are responsible for more than 90% of genital warts^9^. There are currently three prophylactic HPV vaccines on the market: Gardasil (Merck, Sharp and Dohme; Hoddesdon, UK), a quadrivalent vaccine containing HPV 6, 11, 16, and 18 antigens^10^; Gardasil 9 (Merck Sharp and Dohme), a 9-valent vaccine covering HPV 6, 11, 16, 18, 31, 33, 45, 52, and 58 antigens^11^; and Cervarix (GlaxoSmithKline; Brentford, UK), a bivalent vaccine against HPV 16 and 18 antigens^12^.

Cryo-EM structures of virus capsids have shown that the HPV virion assumes a *T* = 7 icosahedral particle containing two capsid proteins, L1 and L2^13,14^. The major capsid protein, L1, can be generated by recombinant expression in insect cells (using baculovirus vectors), yeast cells, plant cells, and *E. coli* cells, and self-assembles into virus-like particles (VLPs) that closely resemble the native papillomavirus virion^15,16^. HPV L1 VLPs are highly immunogenic antigens and can induce the production of high-titer neutralizing antibodies, conferring protection against HPV infection^17,18^. The market-available HPV prophylactic vaccines are all designed based on L1 VLPs and were generated from *Saccharomyces cerevisiae* or insect cells. Other expression systems have also been used to produce HPV L1 proteins, such as *Escherichia coli* (*E. coli*), *Bacillus subtilis*, *Salmonella typhimurium*, silkworm larvae, and plant mammalian cells^19–21^. Among these, *E. coli* has been shown to be an efficient and versatile tool for producing recombinant proteins, offering the advantages of rapid growth rate, inexpensive media for growth, and ease of purification^22–24^. *E. coli* has also been used to express L1 proteins of HPV using GST or β-galactosidase fusion, which helps to alleviate the difficulty associated with the formation of insoluble inclusion bodies^25–30^.

Previous studies on HPV particle assembly have shown that an N-terminally truncated L1 protein lacking over 10 aa could assemble into *T* = 1 small particles, and this suggested that the N-terminal region of the protein was involved in the VLP assembly process^31,32^. Further research found that the 5′-end of the *HPV L1* gene contained an element that could negatively affect the expression of L1 proteins in human epithelial cells^33^, and showed that point mutations or deletions in the inhibitory element to inactivate the effector could improve the production of L1 proteins^24^. However, the exact location of this regulatory region might vary with different HPV types, with maximum protein expression levels achieved with different N-terminal truncations for HPV 6, 11, 16, 18, and 31^34–36^. Therefore, it is necessary to investigate the effects of the N-terminal region on L1 expression for different HPV types for the successful and efficient manufacturing of an *E. coli*-based HPV vaccine.

In our previous work, we successfully produced high-potency VLP vaccine candidates of HPV 6, 11, 16, 18, and 31 using *E. coli*^34–36^, among which the HPV 16/18 bivalent vaccine and HPV 6/11 bivalent vaccine have been in phase 3 and phase 2 clinical trials, respectively. The L1 proteins were produced through non-fusion expression, and harbored N-terminal deletions (except for HPV 31) that rendered them still capable of self-assembling into *T* = 7 particles. Here, we successfully generated L1 VLPs for another four HPV types, HPV 33, 45, 52, and 58, using *E. coli* expression, and found that the non-fusion soluble expression of recombinant HPV 33, 52, and 58 L1 genes could be increased markedly with different N-terminal truncations. We then employed a combination of biochemical and biophysical methods to characterize the structural and antigenic properties of these four VLPs. Combined with our previous L1 VLPs, here we present an *E. coli*-based HPV 9-valent vaccine capable of inducing high and durable neutralizing antibody titers against HPV 6,11,16,18,31,33,45,52,58 infection. Overall, our results introduce a feasible method to optimize the soluble expression of proteins in a bacterial expression system. Furthermore, our 9-valent VLPs generated from *E. coli* offer another highly potent, second-generation, prophylactic HPV vaccine that could benefit the costs associated with worldwide vaccine production and distribution.

## Results

### Design, expression, and purification of HPV L1 proteins with N-terminal truncation

The previously solved structure of the HPV 16 pseudovirus (PDB no:5KEP) highlighted that the initial few amino acids of HPV 16 L1 proteins were not involved in inter-pentameric contacts^37^, suggesting that deletion of these residues might not affect the formation of *T* = 7 particles (Fig. S1). In our previous work, we found that truncation of several N-terminal residues could improve the solubility of HPV L1 proteins^35,36^, and showed that such N-terminal modification offers a feasible strategy to improve the solubility of HPV L1 proteins in *E. coli* for HPV 6, 11, 16, and 18^35,37^. Thus, we further compared the soluble expression levels of L1 proteins among a series of N-terminally truncated L1  constructs of HPV types covered in the commercial HPV 9-valent vaccine (HPV 6, 11, 16, 18, 31, 33, 45, 52, and 58).

**Fig. 1:**
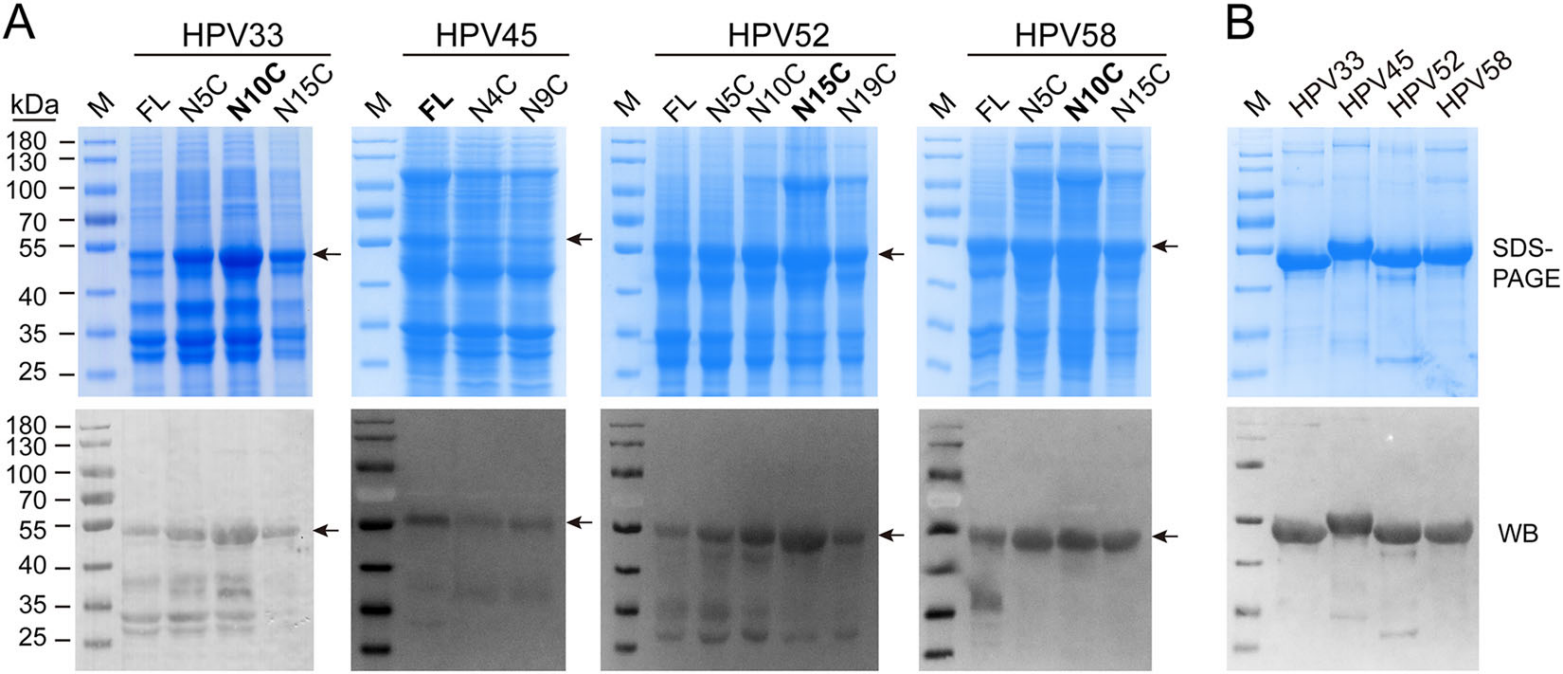
Expression analysis of the N-terminal-truncated L1 proteins and purified L1 proteins of HPV 33, 45, 52, and 58. **a** Whole-cell lysates, expressing different N-terminally truncated L1 proteins of HPV 33, 45, 52, and 58, were subject to SDS-PAGE and western blotting with corresponding type-specific antibodies. L1 proteins are denoted by black arrows. **b** SDS-PAGE and western blotting of purified HPV 33, 45, 52, and 58 L1 proteins. The wide-spectrum HPV L1 linear mAb 4B3 was used to probe the L1 proteins

Full-length gene fragments of HPV 33, 45, 52, and 58 L1 genes were synthesized according to accession numbers GQ479013, DQ080002.1, FJ615303.1, and FJ615305.1, respectively (Fig. S2). Protein expression of full-length and a series of N-terminally truncated HPV 33, 45, 52, and 58 L1 proteins were determined by SDS-PAGE and western blotting (WB) using cell lysates from *E. coli*. (Fig. 1a); we compared these with the five HPV types previously reported (Fig. S3a, S3b). The results showed that, among these nine HPV types, only HPV 31 and 45 showed maximal expression as full-length L1 proteins. By comparison, L1 expression levels of the other HPV types in *E. coli* were increased by N-terminal truncation (Fig. 1a). Taken together, our data indicates that N-terminal residues of the HPV L1 protein affect its soluble expression in *E. coli* to varying degrees, and therefore, it is reasonable to postulate that N-terminal truncation may offer a useful way to optimize protein expression in bacterial systems within a functionally tolerated range.

### Production of a 9-valent vaccine

L1 constructs with the highest protein expression levels—HPV 6 (aa 6–500), HPV 11 (aa 5–501), HPV 16 (aa 5–503), HPV 18 (aa 5–507), HPV 31 (aa 1–504), HPV 33 (aa 10–**4**99), HPV 45 (aa 1–513), HPV 52 (aa 15–503), and HPV 58 (aa 10–498)—were then selected for our 9-valent vaccine. To this end, we scaled up the protein production of truncated HPV 33, 45, 52, and 58 L1 proteins to 5 L-scale fermentation. The purified L1 proteins of HPV 33, 45, 52, and 58 were confirmed for consistency by SDS-PAGE and WB (Fig. 1b). For the purposes of comparison, we also present the results of the purified L1 proteins of HPV 6, 11, 16, 18, and 31 in the Supplementary information (Fig. S3c and S3d). Collectively, the results show that purified L1 proteins of all nine HPV types give prominent bands at an apparent molecular weight of ~55 kDa, and these bands react with an anti-HPV L1 cross-reactive linear mAb (4B3).

### Structural characterization of HPV 33, 45, 52, and 58 L1 VLPs

**Fig. 2:**
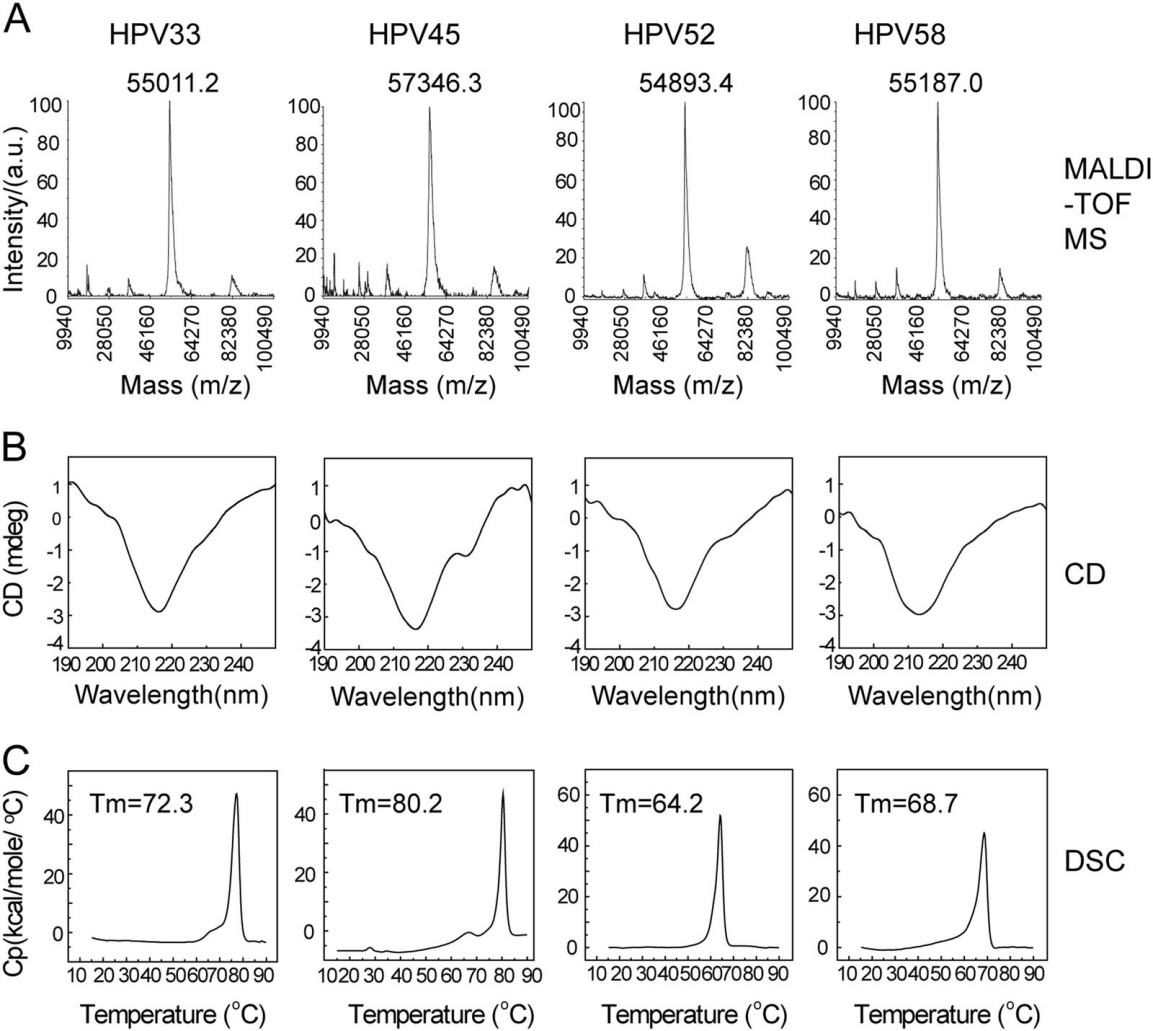
Molecular weight, secondary structure and thermal stability characteristics of the HPV 33, 45, 52, and 58 L1 virus-like particles (VLPs). **a** The molecular weight of HPV L1 proteins was measured using MALDI-TOF MS. The monomer molecular weight of HPV 33, 45, 52, and 58 L1 proteins was ~55–57 kD. **b** Circular dichroism spectra of HPV 33, 45, 52, and 58 L1 virus-like particles (VLPs) showed similar secondary structural compositions of the antigen across different HPV types. **c** Differential scanning calorimetry profiles of HPV L1 proteins show the different transition temperatures. The Tm values of HPV 33, 45, 52, and 58 L1 proteins were 72.3, 80.2, 64.2, and 68.7 °C, respectively

We next used MALDI-TOF MS, circular dichroism spectroscopy (CD) and differential scanning calorimetry (DSC) to test the primary, secondary structures and thermal properties of HPV 33, 45, 52, and 58 L1 proteins. MALDI-TOF MS was employed to determine the intact molecular weight (MW) of HPV 33, 45, 52, and 58 L1 proteins (Fig. 2a). These HPV L1s exhibited similar MWs around 55.0 to 57.3 kDa (HPV 33: 55.0 kDa, HPV 45: 57.3 kDa, HPV 52: 54.9 kDa, and HPV 58: 55.2 kDa), which is consistent with the SDS-PAGE analysis. The CD results showed a similar absorbance curve for HPV 33, 45, 52, and 58 L1 proteins, indicating a similar ratio of secondary structure elements (Fig. 2b). DSC experiments were used to evaluate the thermal stability of the proteins by monitoring the heat capacity during the thermal unfolding process; this is often used to assess the integrity of antigens in a vaccine formulation^38^. The DSC profiles showed only one different thermal transition during particle unfolding for HPV 33, 45, 52, and 58 L1 VLPs at 72.3, 80.2, 64.2, and 68.7 °C, respectively (Fig. 2c).

**Fig. 3:**
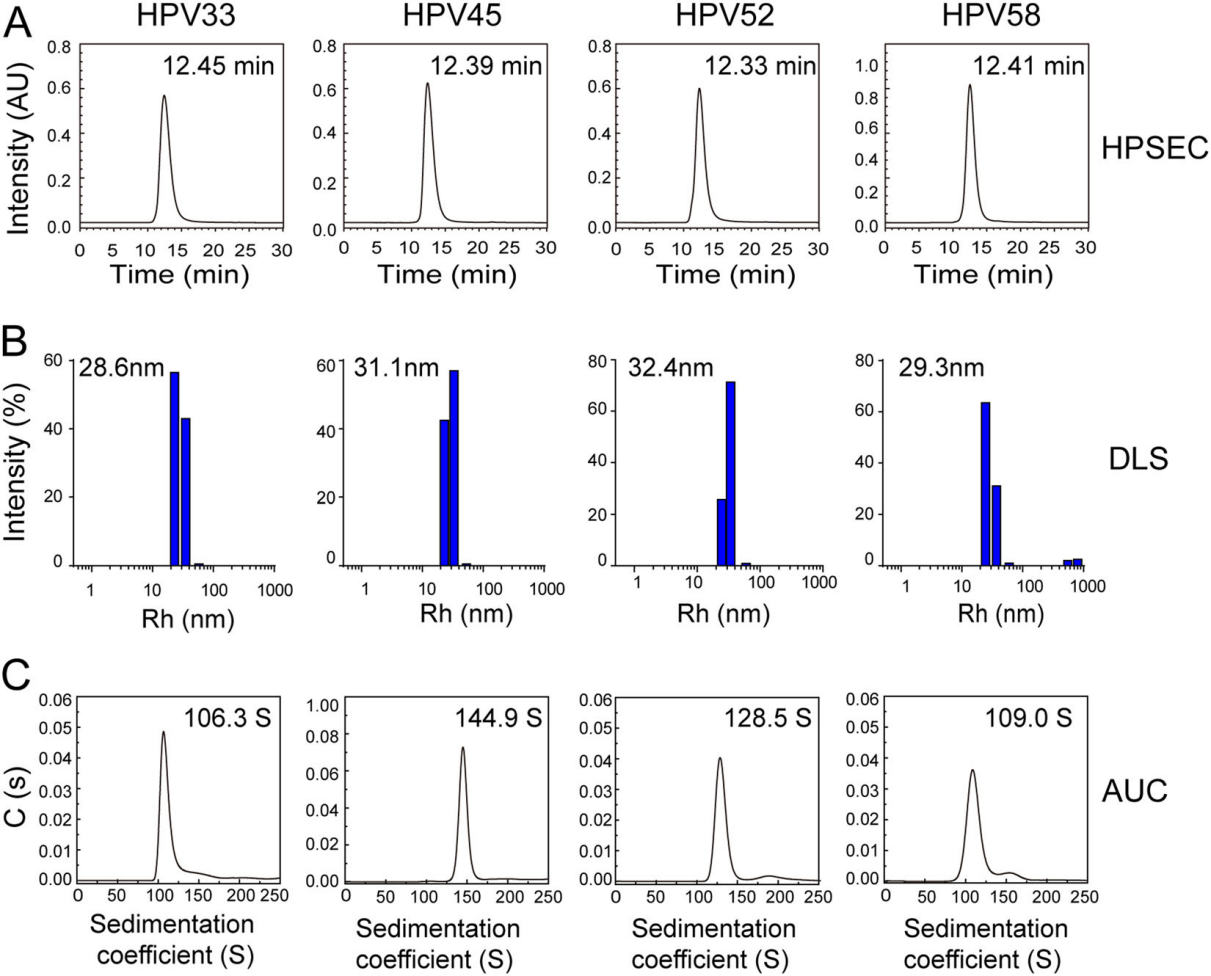
Size and morphology distribution of the HPV 33, 45, 52, and 58 L1 VLPs. **a** High-performance size-exclusion chromatography profiles of HPV 33, 45, 52, and 58 L1 VLPs. **b** Dynamic light scattering analysis of HPV 33, 45, 52, and 58 L1 VLPs. **c** Analytical ultracentrifugation sedimentation profiles of the HPV 33, 45, 52, and 58 L1 VLPs

Purified HPV L1 proteins maintained their pentamer form (about ~8 nm in diameter) under reducing conditions (Fig. S4), and could self-assemble into VLPs in vitro after removing the reducing agent. HPSEC, DLS, and AUC were employed to examine the particle size and sedimentation coefficients of the HPV VLPs. We found that VLPs of HPV 33, 45, 52, and 58 had a similar retention time, ranging from 12.3 to 12.5 min (HPSEC profiles; Fig. 3a). In DLS, the hydrodynamic radii (particles with encircled layers of water molecules) of the main components of the HPV 33, 45, 52, and 58 VLPs were about 28.6, 31.1, 32.4, and 29.3 nm, respectively (Fig. 3b). AUC experiments also showed similar particle properties, with sedimentation coefficients of 106.3S, 144.9S, 128.5S, and 109.0S for HPV 33, 45, 52, and 58 VLPs, respectively (Fig. 3c). Overall, the results indicate that the HPV L1 VLP particle size varies in solution among the different HPV types. The results from particle analyses for HPV 6, 11, 16, 18, and 31 L1 VLP (Fig. S5) were consistent with previous results^34–36^. Overall, the L1 VLPs of nine HPV types qualified for inclusion in the manufactured HPV vaccine.

### Cryo-EM structures of HPV 33, 45, 52, and 58 L1 VLPs

**Fig. 4:**
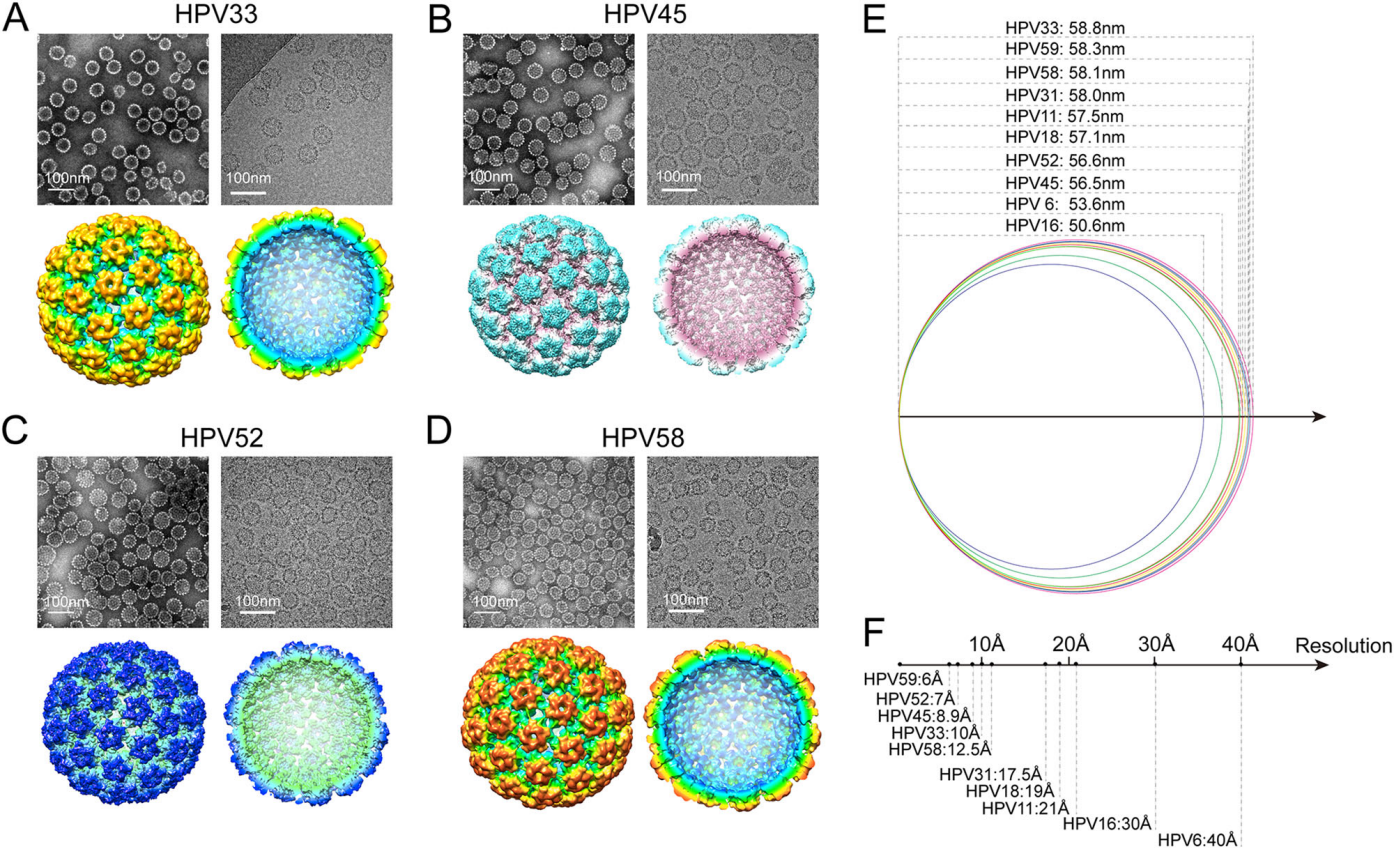
Structural characterization of HPV 33, 45, 52, and 58 L1 particles in solution. (**a–d**, upper) Micrographs of negatively stained (left) and vitrified (right) HPV 33, 45, 52, and 58 L1 VLPs samples. Scale bars, 100 nm; (**a–d**, lower) left, reconstructed 3D cryo-electron microscopy (cryo-EM) maps of HPV 33, 45, 52, and 58 L1 VLPs, color-coded by diameter from 450 to 600 Å, and viewed along the icosahedral twofold axis; right, the same as left but with the closest half of their density map removed to reveal internal features of VLPs of the four types. **e** The diameter distribution of *E. coli*-based L1 VLPs of HPV 6, 11, 16, 18, 31, 33, 45, 52, and 58 measured from their corresponding cryo-EM structures. **f** The resolutions of the cryo-EM reconstructions of *E. coli*-based HPV 6, 11, 16, 18, 31, 33, 45, 52, and 58 L1 VLPs

The morphology of HPV 33, 45, 52, and 58 VLPs was determined by negative staining TEM. Similar to the *E. coli*-generated L1 VLPs for the 5 HPV types determined previously (HPV 6, 11, 16, 18, and 31)^34–36^, particles for HPV 33, 45, 52, and 58 VLPs appeared to be irregularly spherical and heterogeneous in size (Fig. 4a–d, upper left); this was also observed in the unstained, vitrified L1 VLPs (Fig. 4a–d, upper right). Particles were selected from hundreds of low-dose micrographs for HPV 33, 45, 52, and 58, respectively, to reconstruct the 3D structures. With a large set of particles for each type (1,200 to 7,000 particles are used in final reconstruction), we reconstructed cryo-EM structures for all four HPV types at medium-resolution, ranging from ~7 to 12 Å (Fig. 4a–d, lower and Fig. S6). The structures showed that the L1 VLPs for HPV 33, 45, 52, and 58 exhibited *T* = 7 icosahedral particles, with the capsid consisting of 72 pentamers (Fig. 4a–d, lower); this has been observed elsewhere for VLPs generated from different expression systems^35,39,40^. A comparison of the available density maps of all ten HPV types (plus HPV 59)^41^ revealed structural differences in the diameters of the *E. coli*-generated HPV VLPs (HPV 6: 53.6 nm, HPV 11: 57.5 nm, HPV 16: 50.6 nm, HPV 18: 57.1 nm, HPV 31: 58.0 nm, HPV 33: 58.8 nm, HPV 45: 56.5 nm, HPV 52: 56.6 nm, HPV 58: 58.1 nm and HPV 59: 58.3 nm) (Fig. 4e); this may be a consequence of the varied N-terminal truncations that would perhaps normally contribute to the interactions between capsomers during particle assembly^41,42^. It is also worth noting that the map resolution varies a lot among the 10 cryoEM structures (ranging from 6 to 40 Å or lower) (Fig. 4f), which may be possibly caused by the differences in particle homogeneity among the different types. The variance in the dataset sizes used in the reconstructions (~3,000 from 21,000 particles were selected to build a 6 Å HPV 59 VLPs cryoEM structures^41^, whereas only ~1000 particles were used in the final reconstruction of HPV 58 at 12.5 Å) might be another possibility to explain the resolution variance.

### Analysis of the antigenicity and immunogenicity of HPV 9-valent VLPs vaccine

**Fig. 5:**
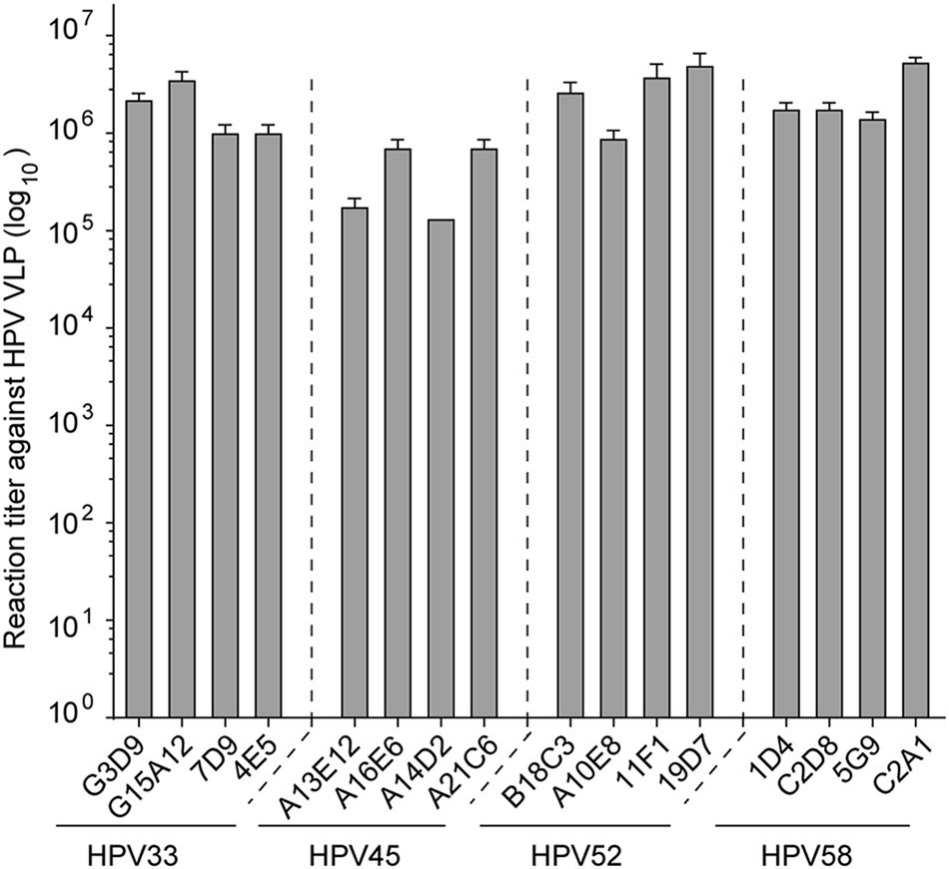
Antigenicity of HPV 33, 45, 52, and 58 L1 VLPs. Binding capacity of mAbs to HPV 33, 45, 52, and 58 L1 VLPs, as determined by ELISA. The reactivities of HPV 33, 45, 52, and 58 L1 VLPs against a panel of genotype-specific neutralizing mAbs were measured by indirect ELISA and are denoted as reactivity titer, which reflects the maximum threefold dilution time to show a positive ELISA reading. All experiments were repeated thrice, and histograms reflect the mean values and standard deviations

We next investigated the antigenicity of HPV L1 VLPs using a conformational mAb panel identified from mice immunized with HPV 33, 45, 52, and 58 VLPs, respectively (Table S1). These mAbs could prevent specific HPV viruses from entering the host cells in a pseudovirus-based neutralization cell model. As shown in Fig. S7, all of the selected mAbs could neutralize the HPV virus of the corresponding type with >10^5^ neutralization titer. We then assessed the antibody binding activity of HPV VLPs using indirect ELISA. We found that HPV 33, 45, 52, and 58 L1 VLPs were capable of binding efficiently (>10^5^ ELISA titer) to their corresponding mAbs (Fig. 5), indicating that HPV 33, 45, 52, and 58 L1 VLPs maintain their native conformational neutralization epitopes, despite the truncation.

We then examined the immunogenicity of the HPV 9-valent vaccine in mice using half-effective dosage (ED_50_) calculations^36^. Mice were separated into groups and vaccinated with the HPV 9-valent vaccine (1.35, 0.45, 0.15, or 0.05 μg; *n* = 10). Sera were collected after 5 weeks to analyze neutralizing antibody titers, and analyzed according to the Reed and Muench statistics^43^. The ED_50_ values for all nine HPV types were lower than 10 ng (7, 4, 6, 3, 2, 2, 3, 2, and 1 ng for HPV 6, 11, 16, 18, 31, 33, 45, 52 and 58, respectively, Table S2).

**Fig. 6:**
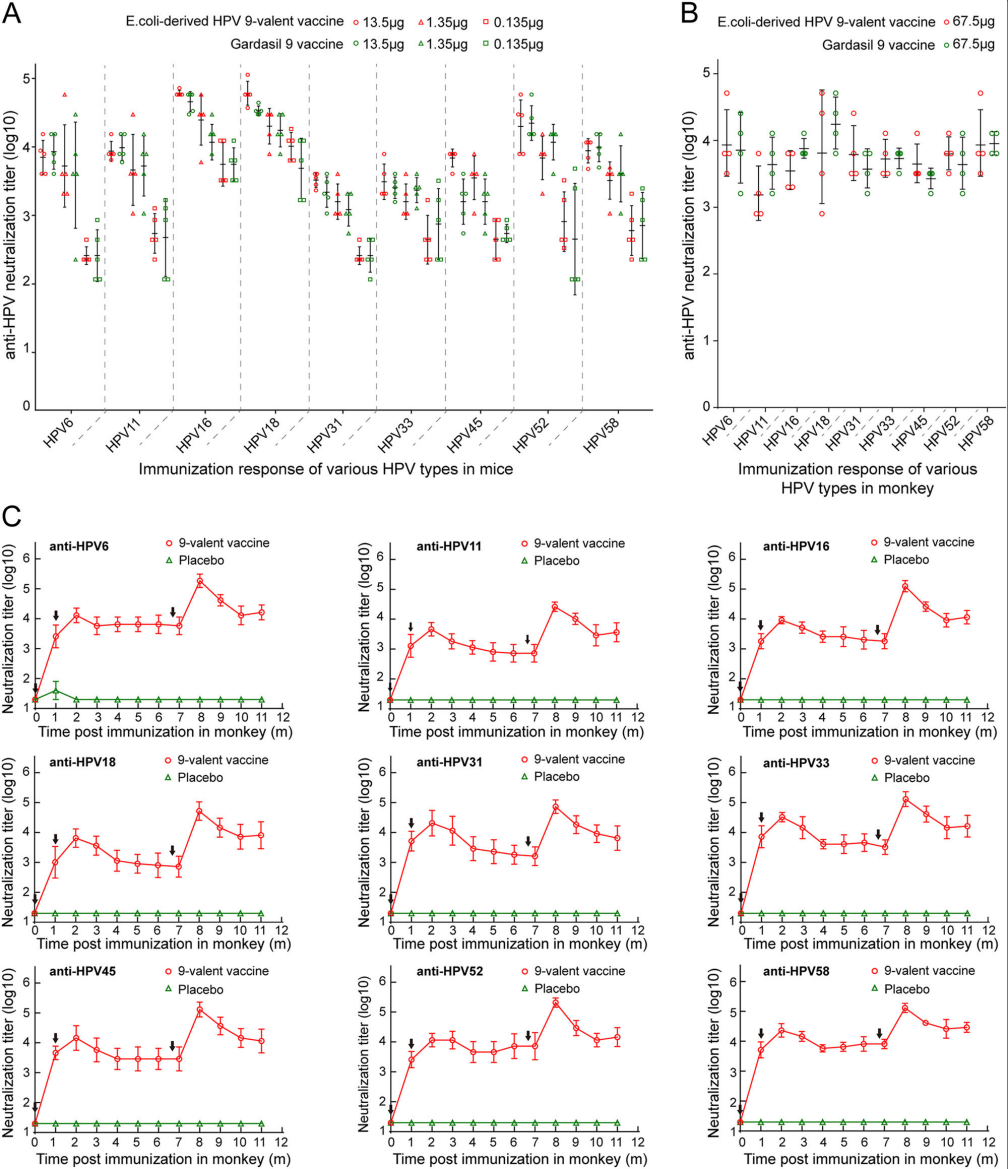
Immunogenicity of HPV 9-valent vaccine. **a**, **b** Comparison of the neutralizing antibody response against HPV 6, 11, 16, 18, 31, 33, 45, 52 and 58 induced by *E. coli*-based HPV 9-valent vaccine with Gardasil 9 vaccines in mice (**a**) and monkeys (**b**). Six groups of mice (*n* = 5) were immunized with 20-, 200-, or 2000-fold diluted dosages of our 9-valent vaccine or Gardasil 9 vaccine at 0, 2, and 4 weeks. Serum samples were collected 2 weeks after the third immunization and titrated using a pseudovirion-based neutralization assay. For monkey trials, two groups of monkeys (*n* *=* 4) were immunized with 1:4 dilution of our 9-valent vaccine or Gardasil 9 vaccine at 0 and 4 weeks. Serum samples collected at 6 weeks post immunization were used to evaluate the neutralization response. **c** Kinetics of serum neutralization titers in Cynomolgus Macaques after immunization with the HPV 9-valent vaccine. Twelve animals were divided into two groups (*n* = 6) and were immunized with 270 μg of HPV 9-valent vaccine and hydroxyl aluminum hydroxide adjuvant as control at 0, 4, and 27 weeks. Immune sera were collected monthly after immunization, and serum neutralization titers were determined and plotted

To compare the immunogenicity of our HPV 9-valent vaccine with Gardasil 9, we immunized six groups of mice (*n* = 5) at 0, 2, and 4 weeks with a 20-, 200- or 2000-fold diluted dosage of our 9-valent vaccine and commercial Gardasil 9^44^. Sera were drawn at 2 weeks after the third vaccination to detect the neutralizing antibody titers. We found that mice receiving as low as 0.135 μg of *E. coli*-generated 9-valent vaccine were capable of inducing about 10^2.36^, 10^2.70^, 10^2.36^, 10^2.60^, 10^2.60^, 10^2.88^, and 10^2.74^ anti-HPV 6, 11, 31, 33, 45, 52, and 58 neutralization titers, and 10^3.75^ and 10^4.02^ against the rest two types, HPV 16 and -18 (Fig. 6a). 1.35 μg dosage led to 10–100-fold higher neutralization titer against nine types, respectively, than 0.135 μg dosage (Fig. 6a). No obvious dose-dependent effect in neutralization response was observed from 1.35 to 13.5 μg (Fig. 6a). Our 9-valent vaccine showed comparable potency against infection of all nine HPV types as that conferred by Gardasil 9 evaluated by antisera immunized by these three different doses (0.135, 1.35, and 13.5 μg). Then, we immunized Cynomolgus Macaques with fourfold diluted dosages of both vaccines (67.5 μg) at 0 and 4 weeks. We had observed that, the anti-HPV 6, 11, 16, 18, 31, 33, 45, 52, and 58 neutralization titers induced by our 9-valent vaccine (Sera were drawn at 2 weeks after the second vaccination) are about 10^3.96^, 10^3.20^, 10^3.55^, 10^3.88^, 10^3.81^, 10^3.73^, 10^3.66^, 10^3.81^, and 10^3.96^, respectively, each of which was capable of reaching to a comparable level with that induced by the commercial vaccine, Gardasil 9 (Fig. 6b).

Next, to investigate the immune persistence of *E. coli*-derived HPV 9-valent vaccine, we immunized Cynomolgus Macaques with 270 μg dosage at 0, 4, and 27 weeks. Similar kinetic curves were observed for neutralizing antibodies against all nine HPV types. At 1 month after the first dose of the vaccine, anti-HPV 6, 11, 16, 18, 31, 33, 45, 52, and 58 neutralization titers were all approaching 10^3^ or higher, and these levels were further enhanced by the second inoculation at 4 weeks. The neutralization titers began to decline slightly and stabilize at titers of above 10^3^ until the third immunization at 27 weeks (Fig. 6c). The maximum titers for HPV 6, 11, 16, 18, 31, 33, 45, 52, and 58—all emerging at 32 weeks (8 months)—were 10^5.3^, 10^4.4^, 10^5.1^, 10^4.7^, 10^4.9^, 10^5.1^, 10^5.1^, 10^5.3^, and 10^5.1^, respectively. The neutralizing antibodies remained high at 17 weeks (~4 months) after the last inoculation, with neutralization titers of 10^4.2^, 10^3.6^, 10^4.1^, 10^3.9^, 10^3.8^, 10^4.2^, 10^4.1^, 10^4.2^, and 10^4.5^, respectively (Fig. 6c). These data demonstrate that our HPV 9-valent vaccine shows high immune efficacy, with excellent durability of the neutralizing antibodies against infection from the nine HPV types.

## Discussion

Several difficulties need to be overcome to successfully express HPV L1 proteins in *E. coli*, such as low expression levels and insoluble inclusion bodies formed by misfolded L1 proteins^21,27,29^. Various strategies, such as using tags for L1 expression^27^ or optimizing expression vectors^28^, have been explored to increase the soluble expression levels of L1 proteins in *E. coli*; however, these approaches have had limited success. Evidence suggests that a region spanning the first 129 nucleotides from the 5′-end of the *L1* gene contains a strong secondary structural inhibitory element that hinders the expression of the L1 protein, and exclusion of such a region could improve the production of L1 proteins in eukaryotic cells^24,33,45^. Our previous *E*. *coli*-based bivalent vaccine candidates, HPV 6/11 and HPV 16/18, also included N-terminally truncated L1 proteins as immunogen^35,36^. In this study, we systematically compared the protein expression levels among different 5′-end-deleted versions of L1 genes of nine HPV types and found that, except for HPV 31 and 45, the full-length L1 proteins showed best expression when truncated (Fig. 1a and S3a), providing further evidence that the expression-sensitive site on the N terminus varies with different HPV types. Thus, optimizing the length of the N-terminal region of L1 proteins without affecting VLP formation could result in high soluble expression levels, which is the perquisite for a successful and efficient *E. coli*-based HPV LI VLPs vaccine.

The SDS-PAGE and MALDI-TOF experiments found similar molecular weights of the HPVs for the nine L1s. All of the L1 proteins in the 9-valent vaccine had the desired characteristics, with similar secondary structure composition, robust thermal stability, and the ability to self-assemble into a VLPs in vitro. Purified HPV 33, 45, 52, and 58 L1 VLPs showed variable particle size but with a mean of ~55 nm, similar to that previously reported^34,46,47^. The heterogeneous sizes of the HPV L1 VLPs among the different types may be caused by the different amino acid sequences of the N-terminal domains, which are important for the assembly of HPV L1 VLPs^27,32^. With respect to the cryoEM reconstructions, we acknowledge that the map resolutions varied considerably among the nine cryoEM structures (ranging from 6 to 40 Å or lower) (Fig. 4f), and this may reflect differences in particle homogeneity among the different types. The varied sizes of the datasets used in the reconstructions (~3,000 particles for the 6-Å HPV 59 VLPs^41^, vs. ~1,000 particles for the 12.5-Å HPV 58) might have also affected the resolution.

By testing the immunogenicity in mice and monkeys, we showed that *E. coli*-based 9-valent vaccine confers comparable neutralization titers against HPV 6, 11, 16, 18, 31, 33, 45, 52, and 58 infection to that induced by Gardasil 9 (Fig. 6a, b), and also indicated that immunized with our 9-valent vaccine was able to provide long-lasting protection against infection of all nine HPV types (Fig. 6c). Thus, our 9-valent vaccine produced in *E. coli* has showed ideal capacity to elicit stable and robust neutralizing antibodies against HPV infection, with a comparable potency to that of Gardasil 9 vaccine.

## Materials and methods

### Plasmids and strain construction

The full-length gene fragments of HPV 33, 45, 52, and 58 L1 (Genbank accession nos: GQ479013, DQ080002.1, FJ615303.1 and FJ615305.1, respectively) were synthesized by GLS (GL Biochem, Shanghai, China). DNA sequences coding for HPV 52 and 58 L1 proteins were from the second initiation codon. The synthesized gene fragments were used as template for PCR amplification, then a series of N-terminally truncated HPV L1 genes were subsequently cloned into *Nde*I/*Sal*I sites in the pTO-T7 expression vector^48^. The *E. coli* strain ER2566 was used to express N-terminally truncated HPV L1 proteins, which were optimized to encode HPV 33 L1 aa 10–499, HPV 45 L1 aa 1–513, HPV 52 L1 aa 15–503, and HPV 58 L1 aa 10–498 (named from the second initiation codon).

### Protein expression, purification, and assembly

N-terminally truncated HPV 33, 45, 52, and 58 L1 proteins were produced as previously described^34,35^. In brief, cells were grown at 37 °C overnight in LB medium and the L1 protein expression was induced by the addition of isopropyl-β-d-thiogalactoside (IPTG, final concentration of 10 μM) upon reaching an OD_600_ of 0.6, and then further incubated at 25 °C for 8 h. Cells were harvested by centrifugation and resuspended with cell lysis solution (20 mM Tris, pH7.4, 300 mM NaCl, and 5 mM EDTA). HPV 33, 45, 52, and 58 L1 proteins were released from the cells by sonication and separated from the cell debris by centrifugation. The supernatant was then combined with 20 mM DTT and further purified by SP sepharose (GE Healthcare, America) and CHT-II resin (Bio-Rad, America). Samples eluted in the 800 mM NaCl fraction. After purification, the proteins were analyzed by SDS-PAGE and dialyzed into neutral buffer (PBS) without a reducing agent to allow VLP self-assembly. The concentration of HPV L1 VLPs was determined using the BCA method.

### Vaccine

Aluminum hydroxide adjuvant was used to absorb HPV 6, 11, 16, 16, 18, 31, 33, 45, 52, and 58 L1 VLPs, respectively. The VLPs were then mixed together. The final product of the HPV 9-valent vaccine contained 30 μg HPV 6 L1 VLPs, 40 μg HPV 11 L1 VLPs, 60 μg HPV 16 L1 VLPs, 40 μg HPV 18 L1 VLPs, 20 μg HPV 31 L1 VLPs, 20 μg HPV 33 L1 VLPs, 20 μg HPV 45 L1 VLPs, 20 μg HPV 52 L1 VLPs, and 20 μg HPV 58 L1 VLPs with 0.42 mg aluminum hydroxide adjuvant suspended in 1.0 mL phosphate-buffered saline (PBS). Gardasil 9 HPV vaccine (Lot no. N023354) was purchased from Hong Kong SAR China.

### Murine monoclonal antibodies (mAbs)

HPV L1-specific mouse mAbs were produced using the hybridoma technology previously described^49,50^. BALB/c mice were immunized subcutaneously three times at 2-week intervals (0, 2, 4 weeks) with nine HPV monovalent vaccines, respectively. The anti-HPV L1 mAbs were produced from mouse ascites fluid and were screened by HPV L1 VLP-based ELISA and a pseudovirion-based neutralization assay. Protein A affinity chromatography was used to purify the anti-HPV mAbs IgGs. The purified mAbs were diluted to 1 mg/mL and stored in PBS at −20 °C.

### SDS-PAGE and western blotting

HPV L1 proteins were analyzed by SDS-PAGE using 10% performed gels. For western blotting, electrophoresed samples transferred to nitrocellulose membranes, were probed with HPV L1 mice antibodies and alkaline phosphatase-conjugated secondary antibody, and scanned as described previously^51^.

### LC–MS and MALDI-TOF MS

According to previously described^38^, HPV 33, 45, 52, and 58 VLPs were treated using reducing and alkylating conditions before tryptic digestion. The peptide fragments and their amino acid compositions were analyzed using the Triple TOF 5600 System fitted with a Nanospray III source (AB SCIEX, Concord, Ontario, Canada) and a pulled quartz tip as the emitter (New Objectives, Woburn, MA). The mass spectra of HPV L1 peptide mixtures were gained using a Reflex III MALDI-TOF mass spectrometer (Burker Daltonik, Bremen, Germany) with α-cyano-4-hydroxy cinnamic acid as a matrix before the MS analysis.

### Circular dichroism (CD)

CD spectra were acquired using a Jasco J-810 spectropolarimeter (Jasco Inc., Easton, MD), as previously described by Zhang et al.^38^ HPV 33, 45, 52, and 58 VLPs were placed into a 1.0-mm path-length cuvette for far-UV measurements.

### Thermal stability by differential scanning calorimetry (DSC)

The thermostability of HPV 33, 45, 52, and 58 VLPs was determined using a MicroCal VP-DSC instrument (GE Healthcare, MicroCal Products Group, Northampton, MA), as previously described by Zhang et al.^38^

### Particle size analysis

Particle size is an essential parameter of a VLP-based vaccine. The distribution of molecular sizes, homogeneity and sedimentation velocity, hydrodynamic size distributions and overall morphology distribution of purified HPV 33, 45, 52, and 58 VLPs were investigated using size-exclusion chromatography (HPSEC), analytical ultracentrifugation (AUC), dynamic light scattering (DLS), and transmission electron micrograph (TEM), respectively, as previously described^35^.

### Cryo-electron microscopy (Cryo-EM)

As described by Li et al.^41^, purified HPV 33, 45, 52, and 58 VLPs, at a concentration of ~3 mg/mL, were vitrified on Quantifoil holey carbon grids in an FEI Vitrobot. Images were recorded on an FEI Falcon II direct detector camera at a nominal ×93,000 magnification in an FEI TF30 FEG microscope at 300 kV, with underfocus settings estimated to be between 1 and 3 μm, and with an electron dose of 25 e/Å^2^. The *T* = 7 particle images were manually boxed and extracted with the program Robem^52^. RELION, AUTO3DEM, and EMAN2 programs were used for image processing and three-dimensional (3D) reconstructions^53,54^.

### Indirect binding ELISA

The antigenicity of HPV 33, 45, 52, and 58 VLPs was determined by indirect binding ELISA, as described by Pan et al.^35^ HPV L1 VLPs were coated into the wells of 96-well microplates (100 ng/well). The wells were blocked and then incubated with 100 μL of twofold serially diluted anti-HPV 33, 45, 52, and 58 L1 mAbs. HRP-conjugated goat anti-mouse Ig antibody (diluted 1:5000 in HS-PBS, Abcam; Cambridge, UK) was used as a secondary antibody. The absorbance (450 nm; reference, 620 nm) was recorded using an automated ELISA reader (TECAN, Männedorf, Switzerland). The cut-off value was set at an absorbance of 450 (Δ620) nm = 0.1 to define the positive titer.

### Animal study

For ED_50_ calculation assays, the final HPV 9-valent vaccine was diluted 200-fold, 600-fold, 1,800-fold, 5,400-fold, and 16,200-fold with aluminum hydroxide adjuvant. Fifty special pathogen-free (SPF) BALB/c mice (*n* = 10 per group) were vaccinated intra-muscularly with a single dose of HPV 9-valent vaccine. Serum samples were taken 5 weeks after immunization to determine the seroconversion of anti-HPV 6, 11, 16, 16, 18, 31, 33, 45, 52, or 58 neutralizing antibodies.

The immunogenicity of the HPV 9-valent vaccine was tested in mice and adult Cynomolgus Macaques. For mice immunizations, 4- to 6-week-old female BLAB/c mice were immunized three times at 0, 2, and 4 weeks with intraperitoneal injections of a 1:20, 1:200, or 1:2,000 dilution of HPV 9-valent vaccine, respectively, and Gardasil 9 vaccine served as controls. The sera were harvested at week 6 and subjected to neutralization assay. For the non-human primate study, a total of eight monkeys were used during screening. These monkeys showed no detectable anti-HPV L1 IgG in serum at 1:10 dilution using ELISA testing. To compare the immunogenicity of our *E. coli*-based HPV 9-valent vaccine with Gardasil 9 vaccines, two groups (*n* = 4) monkeys were immunized three times with a 1:4 dilution of HPV 9-valent vaccine or Gardasil 9 vaccine at weeks 0 and 4. The serum samples were collected at 6-week post first immunization to detect anti-HPV L1 neutralizing antibodies. To assess the kinetics of virus-neutralizing antibody induced by our *E. coli*-based HPV 9-valent vaccine, the other two groups monkeys (*n* = 6) were immunized three times at weeks 0, 4, and 27 with an intramuscular injection of 270 μg HPV 9-valent vaccine; the placebo group received aluminum hydroxide adjuvant only. Serum samples were collected weekly after the first immunization to identify anti-HPV 6, 11, 16, 18, 31, 33, 45, 52, and 58 neutralizing antibodies in a pseudovirus-binding neutralizing assay.

### Pseudovirus-based neutralization assay

HPV 6, 11, 16, 18, 31, 33, 45, 52, and 58 pseudoviruses were produced according to previous studies^55–57^. HPV 6,11,16,18 L1/L2 expression vector and pN31-EGFP used in the experiment were kindly provided by Dr. J. T. Schiller^58^. HPV 31, 33, 45, 52, and 58 L1/L2 genes were chemically synthesized by GLS (GL Biochem, Shanghai, China). In briefly, 293FT cell were harvested 72 h after transfection, lysed with cell lysis buffer containing 0.5% Brij58 (Sigma-Aldrich; St Louis, MO), 0.2% Benzonase (Merck Millipore; Darmstadt, Germany), and 0.2% PlasmidSafe ATP-Dependent DNase (Epicenter Biotechnologies, Madison, WI) DPBS-Mg solution, and incubated at 37 °C for 24 h. Samples were then combined with a 5 M NaCl solution to extract the cell lysates. The TCID_50_ (tissue culture infective dose) of the supernatants was then measured to determine the titers of the PsVs, calculated according to the classical Reed–Muench method^59^.

A pseudovirus-binding neutralizing assay was used to determine anti-HPV L1 neutralizing antibodies within the samples, as previously described^56,57^. Briefly, 293FT cells were incubated at 37 °C in the wells of a 96-well plate at a density of 1.5 × 10^4^ cells per well for 6 h. The sera of mouse and monkeys were 2-fold diluted, and PsVs were diluted to 3 × 10^5^ TCID_50_/µL. Equal volumes (60 µL) of the PsV diluent and the serially diluted sera were mixed and incubated at 4 °C for 1 h. The negative control was prepared by mixing equal volumes (60 µL) of the PsV diluent and culture medium. Then, 100 µL of these mixtures were added to designated wells and incubated at 37 °C for 72 h. After 72 h, the cells were trypsinized and analyzed by flow cytometry. The endpoint titers were calculated as the log_10_ of the highest sera or antibody dilution with a percent infection inhibition higher than 50%. Every sample was detected at least three times, and the values presented here are calculated as the mean value of all repeats.

### Data bank accession numbers

The EM density map for HPV 33, 45, 52, and 58 VLPs have been deposited in the Electron Microscopy DataBank (EMDB: EMD-6909, EMD-6919, EMD-6918, and EMD-6910, respectively).

## Electronic supplementary material

Supplementary information
