# Supplementary material for: N-terminal truncations on L1 proteins of human papillomaviruses promote their soluble expression in Escherichia coli and self-assembly in vitro
Source: Emerg Microbes Infect. 2018 Sep 26;7:160. doi: 10.1038/s41426-018-0158-2 (PMC6156512; doi:10.1038/s41426-018-0158-2)
Supplement: Supplementary file 1 — Supplementary information [file 41426_2018_158_MOESM1_ESM.docx]

**Supplementary information**


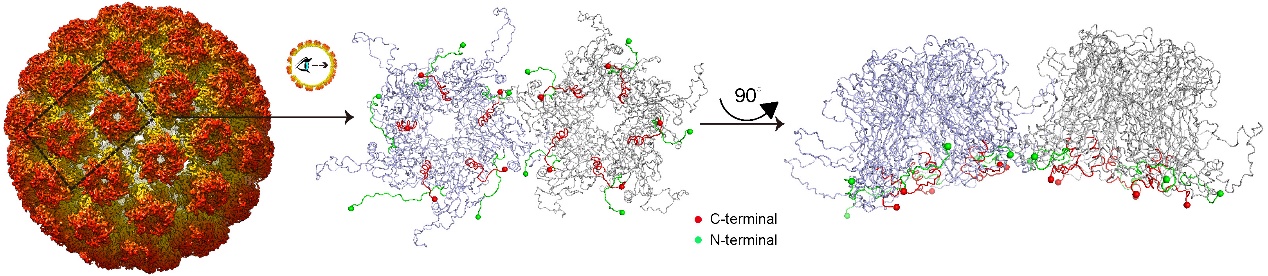


**Supplementary Figure 1.** Model representation to show the N/C- terminal regions of HPV16 L1 particle (PDB no:5KEP) ([1](#_ENREF_1)). N-terminal regions colored in green, C-terminal regions colored in red.


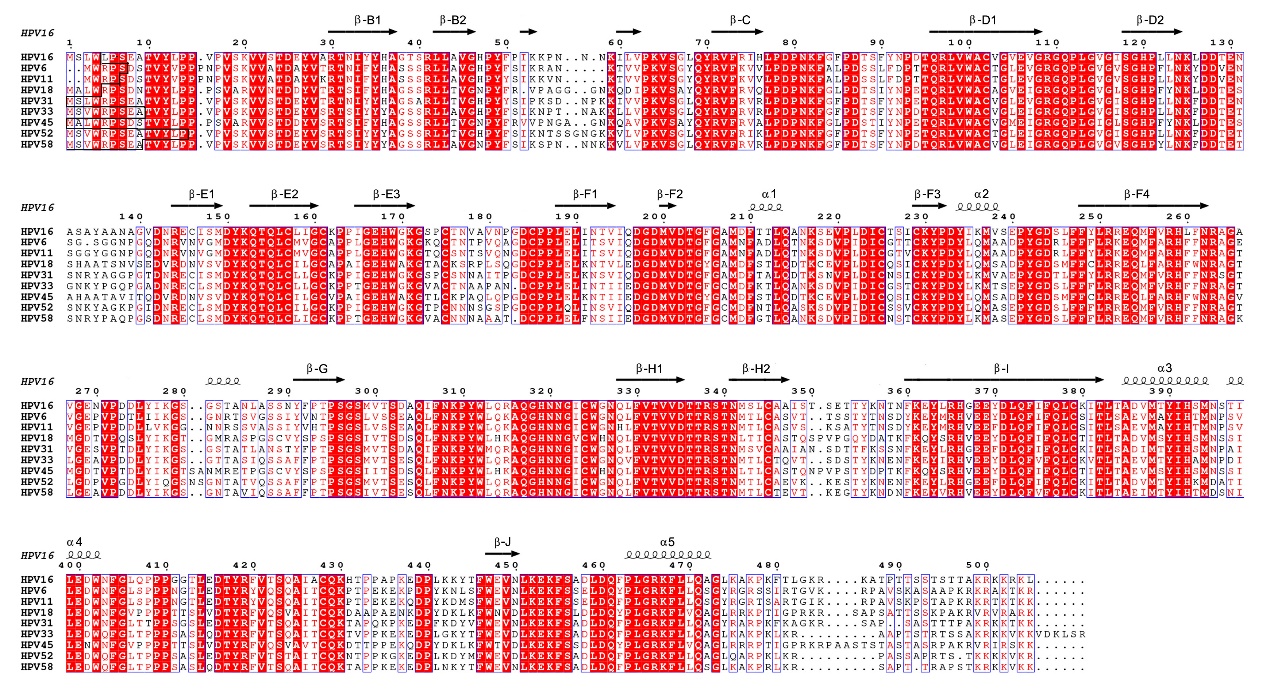


**Supplementary Figure 2.** Sequence alignments of the L1 proteins 9 HPV serotypes. Secondary structural elements are shown above the sequences. Alignments were determined and plotted using Chimera([2](#_ENREF_2)).


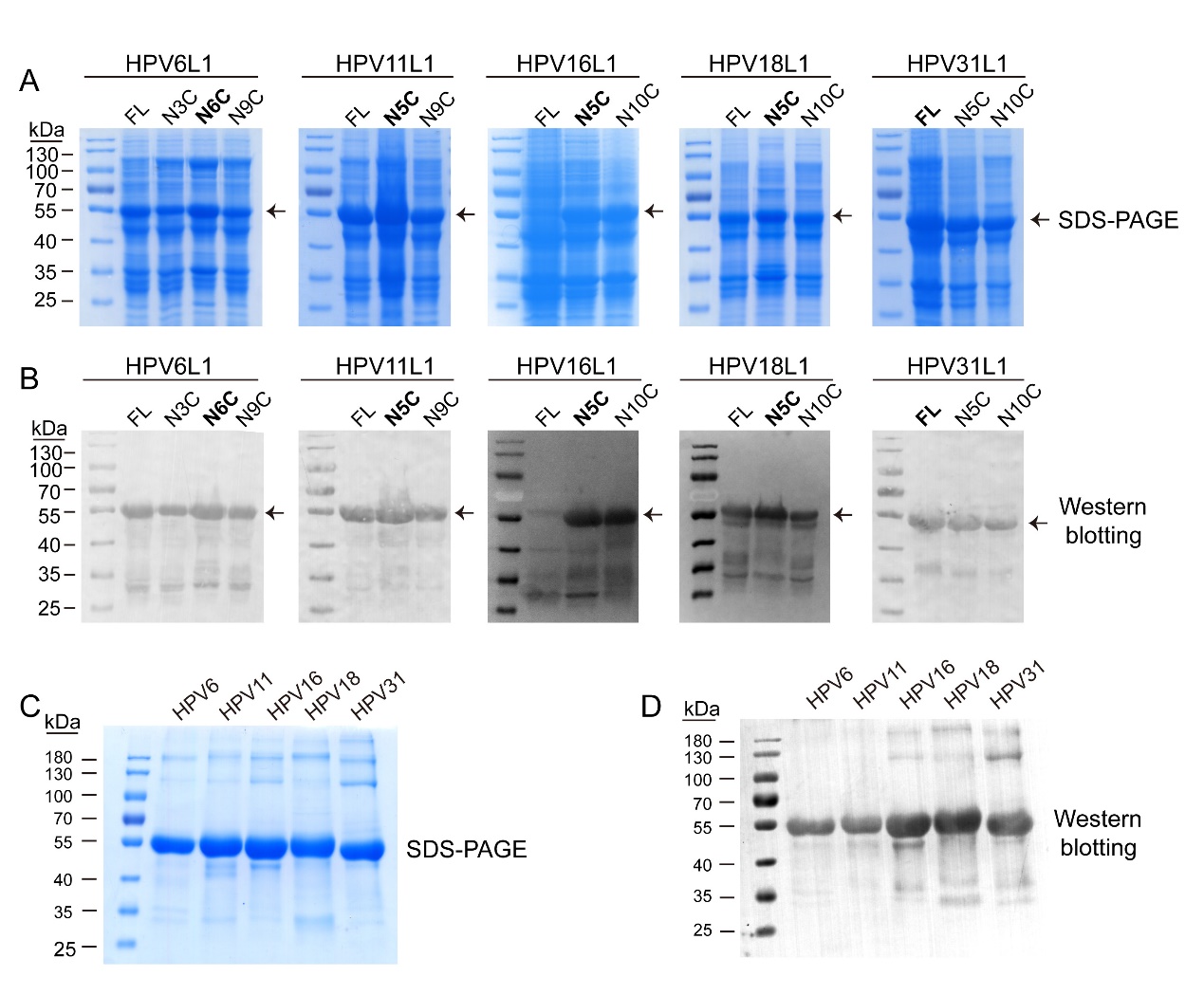


**Supplementary Figure 3.** Expression analysis of the N-terminal truncated L1 proteins and purified L1 proteins of HPV 6, 11, 16, 18 and 31. (A-B) Whole cell lysates, expressing different N-terminally truncated L1 proteins of HPV 6, 11, 16, 18 and 31, were subject to SDS-PAGE (A) and western blotting with corresponding type-specific antibodies (B). L1 proteins are denoted by black arrows. (C) SDS-PAGE and (D) western blotting of purified HPV 6, 11, 16, 18 and 31 L1 proteins. The wide-spectrum HPV L1 linear mAb 4B3 was used to probe the L1 proteins.


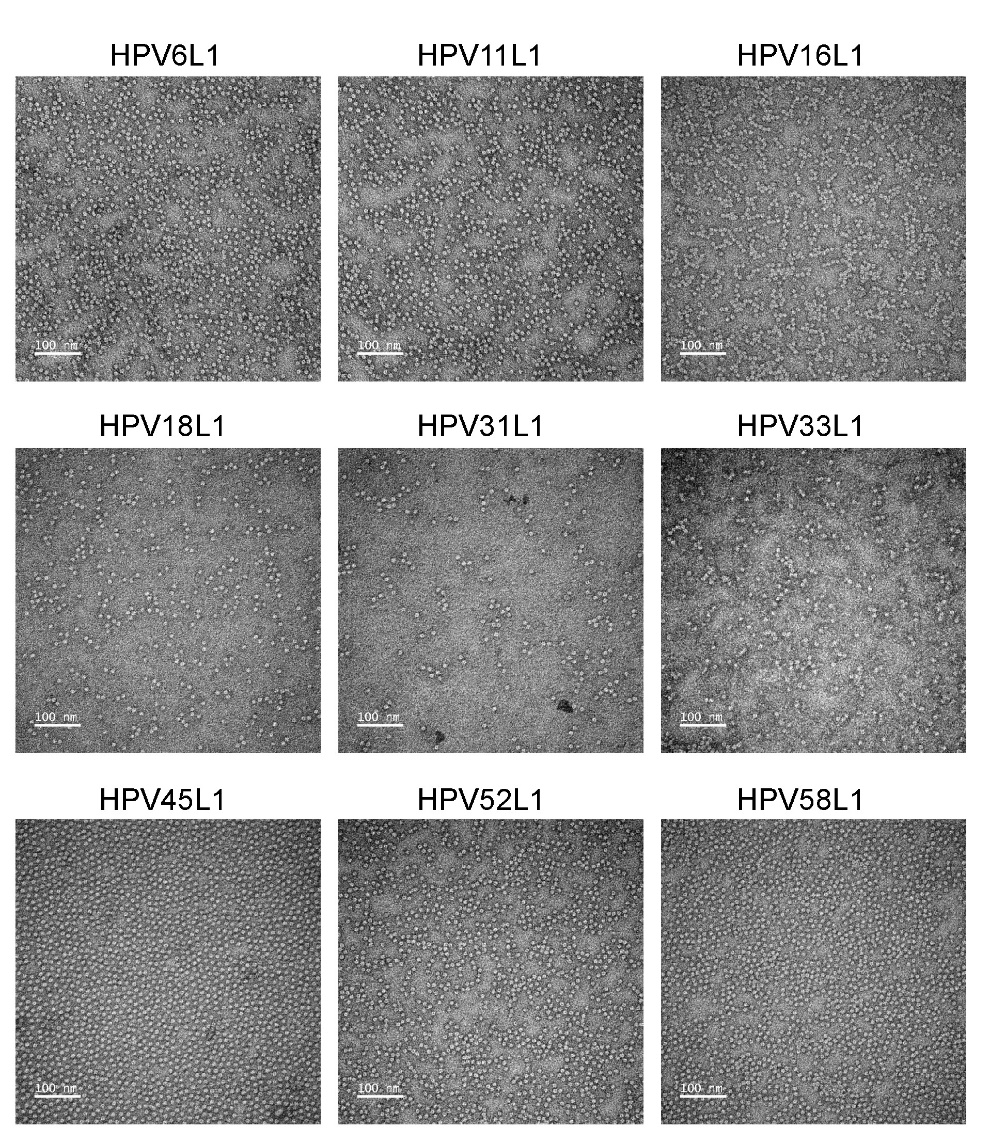


**Supplementary Figure 4.** Transmission electron microscopy (TEM) images of the purified HPV 6, 11, 16, 18, 31, 33, 45, 52 and 58 L1 proteins which were retention in reducing condition. Bar, 100nm.


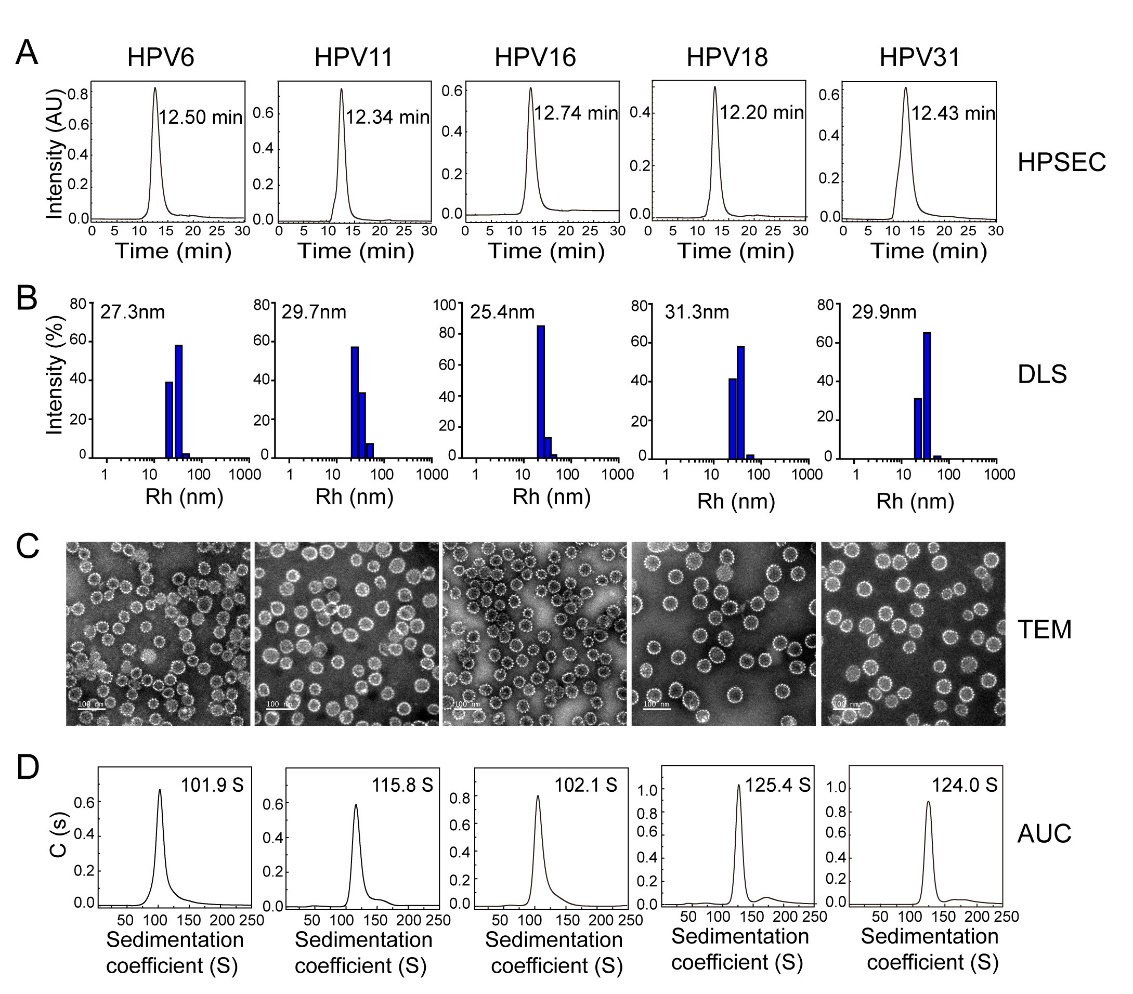


**Supplementary Figure 5.** Size and morphology distribution of the HPV 6, 11, 16, 18 and 31 L1 VLPs. (A) High-performance size-exclusion chromatography profiles of HPV 6, 11, 16, 18 and 31 L1 VLPs. (B) Dynamic light scattering analysis of HPV 6, 11, 16, 18 and 31 L1 VLPs. (C) Micrographs of negatively stained HPV 6, 11, 16, 18 and 31 L1 VLPs samples. Scale bars, 100 nm (D) Analytical ultracentrifugation sedimentation profiles of the HPV 6, 11, 16, 18 and 31 L1 VLPs.


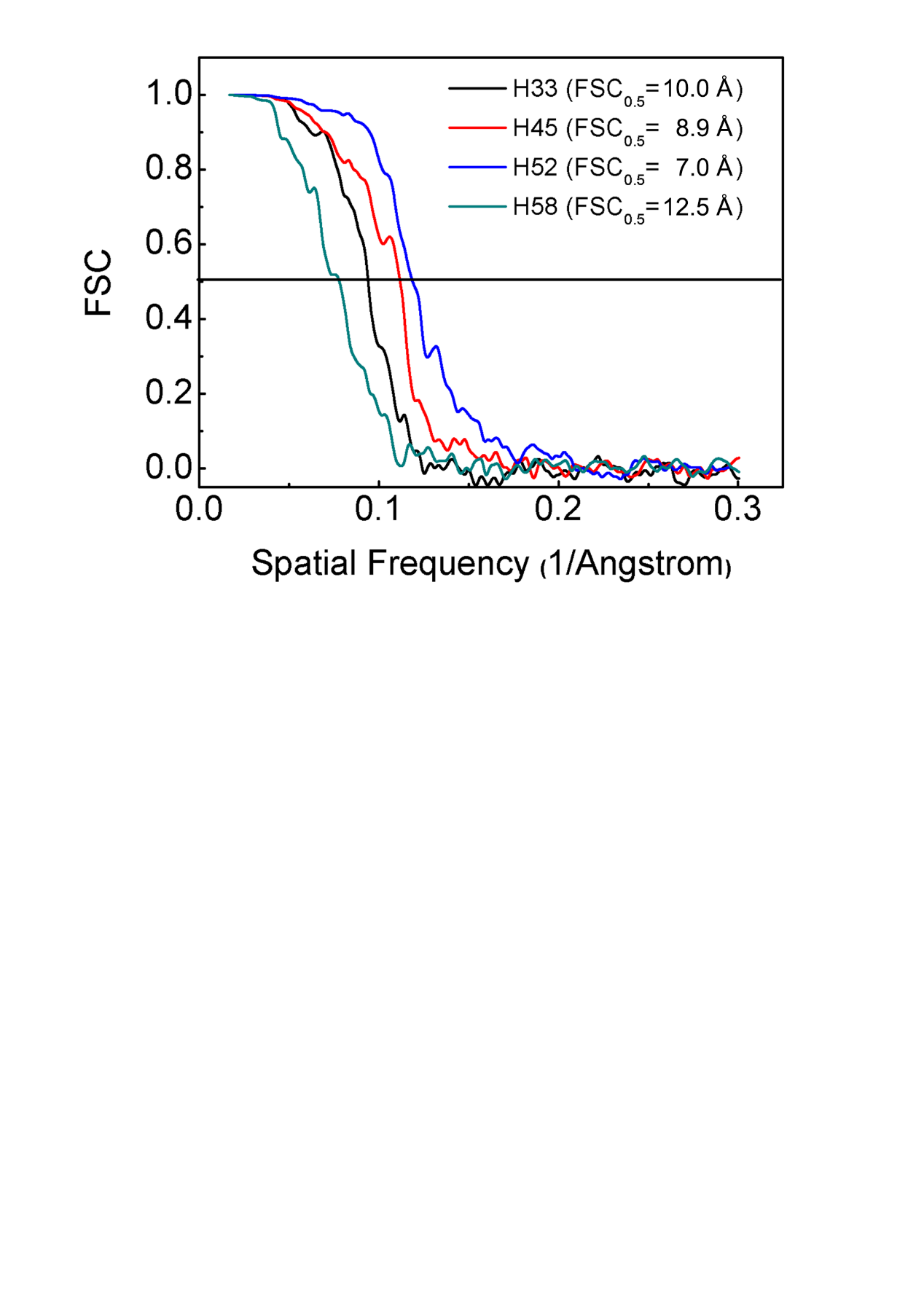


**Supplementary Figure 6.** Resolution evaluation of the cryo-EM reconstructions by Fourier shell correlation (FSC) plot of the HPV33, 45, 52 and 58 L1 VLP cryo-reconstruction. Resolution evaluation of the cryo-EM reconstructions by FSC at 0.5 criterion.


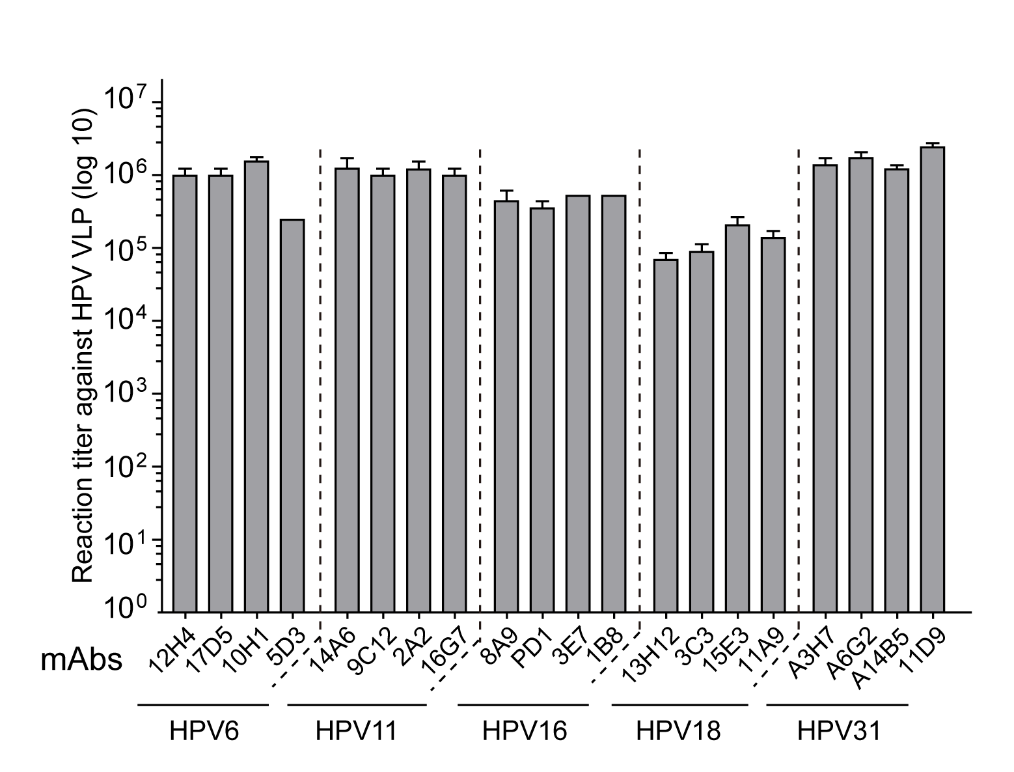


**Supplementary Figure 7.** Antigenicity of HPV 6, 11, 16, 18 and 31 L1 VLPs. Binding capacity of mAbs to HPV 6, 11, 16, 18 and 31 L1 VLPs, as determined by ELISA. The reactivities of HPV 6, 11, 16, 18 and 31 L1 VLPs against a panel of genotype-specific neutralizing mAbs were measured by indirect ELISA and are denoted as reactivity titer, which reflects the maximum 3-fold dilution time to show a positive ELISA reading. All experiments were repeated thrice, and histograms reflect the mean and standard errors.

**Supplementary Table 1**. Screen and characterization of anti-HPV6, 11, 16, 18, 31, 33, 45, 52 and 58 neutralizing monoclonal antibodies for relative antigenicity assay of HPV nine-valent vaccine

| Immunogen | mAbs | Isotype | Conformation of  linear (C or L) | Neutralizing  titer |
| --- | --- | --- | --- | --- |
| HPV 6 VLPs | 12H4 | IgG1 | C | 10^6^ |
|  | 17D5 | IgG2a | C | 10^5^ |
|  | 10H1 | IgG1 | C | 10^8^ |
|  | 5D3 | IgG2b | C | 10^5^ |
| HPV 11 VLPs | 14A6 | IgG1 | C | 10^5^ |
|  | 9C12 | IgG1 | C | 10^5^ |
|  | 2A2 | IgG1 | C | 10^4^ |
|  | 16G7 | IgG1 | C | 10^5^ |
| HPV 16 VLPs | 8A9 | IgG2b | C | 10^5^ |
|  | PD4 | IgG2a | C | 10^5^ |
|  | 3E7 | IgG2b | C | 10^5^ |
|  | 1D12 | IgG2b | C | 10^4^ |
| HPV 18 VLPs | 13H12 | IgG2b | C | 10^5^ |
|  | 3C3 | IgG2b | C | 10^5^ |
|  | 15E3 | IgG1 | C | 10^3^ |
|  | 11A9 | IgG2a | C | 10^5^ |
| HPV 31 VLPs | A3H7 | IgG2b | C | 10^5^ |
|  | A6G2 | IgG2b | C | 10^6^ |
|  | A14B5 | IgG2b | C | 10^6^ |
|  | 11D9 | IgG1 | C | 10^5^ |
| HPV 33 VLPs | G3D9 | IgG2b | C | 10^6^ |
|  | G15A12 | IgG2b | C | 10^6^ |
|  | 7D9 | IgG1 | C | 10^5^ |
|  | 4E5 | IgG1 | C | 10^5^ |
| HPV 45 VLPs | A13E12 | IgG2b | C | 10^5^ |
|  | A16E6 | IgG2a | C | 10^5^ |
|  | A14D2 | IgG2b | C | 10^5^ |
|  | A21C6 | IgG2b | C | 10^5^ |
| HPV 52 VLPs | B18C3 | IgG2b | C | 10^6^ |
|  | A10E8 | IgG1 | C | 10^5^ |
|  | 11F1 | IgG2b | C | 10^6^ |
|  | 19D7 | IgG2a | C | 10^5^ |
| HPV 58 VLPs | 1D4 | IgG2b | C | 10^6^ |
|  | C2D8 | IgG2a | C | 10^6^ |
|  | 5G9 | IgG2b | C | 10^6^ |
|  | C2A1 | IgG2b | C | 10^5^ |

**Supplementary** **Table 2.** The half effective dosage (ED_50_) of HPV6, 11, 16, 18, 31, 33, 45, 52, 58 nine-valent vaccine

| HPV 9-valent vaccine (μg) | Antigen  (VLP) | Dose  (μg) | Seroconvertion no./  inoculated no (Positve rate) | | | ED_50_ (μg)* |
| --- | --- | --- | --- | --- | --- | --- |
| 1.35μg | HPV6 VLP | 0.15 | | 10/10 (100%) | 0.007 | |
| 0.45μg |  | 0.05 | | 10/10 (100%) |  |  |
| 0.15μg |  | 0.017 | | 10/10 (100%) |  |  |
| 0.05μg |  | 0.006 | | 4/10 (40%) |  |  |
| 0.016μg |  | 0.002 | | 0/10 (0%) |  |  |
| 1.35μg | HPV11 VLP | 0.2 | | 10/10 (100%) | 0.004 | |
| 0.45μg |  | 0.067 | | 10/10 (100%) |  |  |
| 0.15μg |  | 0.022 | | 10/10 (100%) |  |  |
| 0.05μg |  | 0.007 | | 9/10 (91.67%) |  |  |
| 0.016μg |  | 0.002 | | 2/10 (18.18%) |  |  |
| 1.35μg | HPV16 VLP | 0.3 | | 10/10 (100%) | 0.006 | |
| 0.45μg |  | 0.1 | | 10/10 (100%) |  |  |
| 0.15μg |  | 0.033 | | 10/10 (100%) |  |  |
| 0.05μg |  | 0.011 | | 10/10 (100%) |  |  |
| 0.016μg |  | 0.004 | | 1/10 (10%) |  |  |
| 1.35μg | HPV18 VLP | 0.2 | | 10/10 (100%) | 0.003 | |
| 0.45μg |  | 0.067 | | 10/10 (100%) |  |  |
| 0.15μg |  | 0.022 | | 10/10 (100%) |  |  |
| 0.05μg |  | 0.007 | | 9/10 (92.86%) |  |  |
| 0.016μg |  | 0.002 | | 4/10 (36.36%) |  |  |
| 1.35μg | HPV31 VLP | 0.1 | | 10/10 (100%) | 0.002 | |
| 0.45μg |  | 0.033 | | 10/10 (100%) |  |  |
| 0.15μg |  | 0.011 | | 10/10 100%) |  |  |
| 0.05μg |  | 0.003 | | 9/10 (90.91%) |  |  |
| 0.016μg |  | 0.001 | | 1/10 (9.09%) |  |  |
| 1.35μg | HPV33 VLP | 0.1 | | 10/10 (100%) | 0.002 | |
| 0.45μg |  | 0.033 | | 10/10 (100%) |  |  |
| 0.15μg |  | 0.011 | | 10/10 (100%) |  |  |
| 0.05μg |  | 0.003 | | 9/10 (92.31%) |  |  |
| 0.016μg |  | 0.001 | | 3/10 (27.27%) |  |  |
| 1.35μg | HPV45 VLP | 0.1 | | 10/10 (100%) | 0.003 | |
| 0.45μg |  | 0.033 | | 10/10 (100%) |  |  |
| 0.15μg |  | 0.011 | | 7/10 (82.35%) |  |  |
| 0.05μg |  | 0.003 | | 7/10 (53.85%) |  |  |
| 0.016μg |  | 0.001 | | 0/10 (0%) |  |  |
| 1.35μg | HPV52 VLP | 0.1 | | 10/10 (100%) | 0.02 | |
| 0.45μg |  | 0.033 | | 10/10 (100%) |  |  |
| 0.15μg |  | 0.011 | | 10/10 (100%) |  |  |
| 0.05μg |  | 0.003 | | 8/10 (81.82%) |  |  |
| 0.016μg |  | 0.001 | | 1/10 (8.33%) |  |  |
| 1.35μg | HPV58 VLP | 0.1 | | 10/10 (100%) | 0.001 | |
| 0.45μg |  | 0.033 | | 10/10 (100%) |  |  |
| 0.15μg |  | 0.011 | | 10/10 (100%) |  |  |
| 0.05μg |  | 0.003 | | 10/10 (100%) |  |  |
| 0.016μg |  | 0.001 | | 4/10 (40%) |  |  |

BALB/c mice were [intraperitoneal](http://www.baidu.com/link?url=Vds1-NgTPM0tJC-JDxipIggmmMWkgAbnWm7ltmZUCUJK77yKk0Ewn1DW_y7RCcVKQyl_bjRdzbnEuVeuk6Txf2pfaNXwwoJNJGRKxBMY6FcmFVmJ0NNZ_0H-hOAk7QRy)ly inoculated once with the indicated doses of HPV6, 11, 16, 18, 31, 33, 45, 52 or 58 vaccine and bled 4 weeks later for antibody response. VLPs were formulated with aluminum adjuvant.

* The ED_50_ values for HPV6 6, 11, 16, 18, 31, 33, 45, 52 and 58 were calculated according to the method by Reed and Muench analysis ([3](#_ENREF_3)).

**Supplementary References**

1. Guan, J. et al. Cryoelectron Microscopy Maps of Human Papillomavirus 16 Reveal L2 Densities and Heparin Binding Site. *Structure* **25**, 253-263 (2017).

2. Pettersen, E. F. et al. UCSF Chimera--a visualization system for exploratory research and analysis. *Journal of computational chemistry* **25**, 1605-1612 (2004).

3. Reed LJ, M. H. A simple method of estimating fifty percent endpoints. *Am J Hyg* **27**, 493-497 (1938).
